# Population Structure, Genetic Variation, and Linkage Disequilibrium in Perennial Ryegrass Populations Divergently Selected for Freezing Tolerance

**DOI:** 10.3389/fpls.2015.00929

**Published:** 2015-11-12

**Authors:** Mallikarjuna Rao Kovi, Siri Fjellheim, Simen R. Sandve, Arild Larsen, Heidi Rudi, Torben Asp, Matthew Peter Kent, Odd Arne Rognli

**Affiliations:** ^1^Department of Plant Sciences, Centre for Integrative Genetics, Norwegian University of Life SciencesÅs, Norway; ^2^Department of Animal and Aquacultural Sciences, Centre for Integrative Genetics, Norwegian University of Life SciencesÅs, Norway; ^3^Graminor AS, TorggårdenBodø, Norway; ^4^Department of Chemistry, Biotechnology and Food Science, Norwegian University of Life SciencesÅs, Norway; ^5^Department of Molecular Biology and Genetics, Aarhus UniversitySlagelse, Denmark

**Keywords:** freezing tolerance, population structure, linkage disequilibrium, genetic diversity, outlier SNPs, *Lolium perenne*

## Abstract

Low temperature is one of the abiotic stresses seriously affecting the growth of perennial ryegrass (*Lolium perenne L.*), and freezing tolerance is a complex trait of major agronomical importance in northern and central Europe. Understanding the genetic control of freezing tolerance would aid in the development of cultivars of perennial ryegrass with improved adaptation to frost. The plant material investigated in this study was an experimental synthetic population derived from pair-crosses among five European perennial ryegrass genotypes, representing adaptations to a range of climatic conditions across Europe. A total number of 80 individuals (24 of High frost [HF]; 29 of Low frost [LF], and 27 of Unselected [US]) from the second generation of the two divergently selected populations and an unselected (US) control population were genotyped using 278 genome-wide SNPs derived from perennial ryegrass transcriptome sequences. Our studies investigated the genetic diversity among the three experimental populations by analysis of molecular variance and population structure, and determined that the HF and LF populations are very divergent after selection for freezing tolerance, whereas the HF and US populations are more similar. Linkage disequilibrium (LD) decay varied across the seven chromosomes and the conspicuous pattern of LD between the HF and LF population confirmed their divergence in freezing tolerance. Furthermore, two *F*_*st*_ outlier methods; finite island model (fdist) by LOSITAN and hierarchical structure model using ARLEQUIN, both detected six loci under directional selection. These outlier loci are most probably linked to genes involved in freezing tolerance, cold adaptation, and abiotic stress. These six candidate loci under directional selection for freezing tolerance might be potential marker resources for breeding perennial ryegrass cultivars with improved freezing tolerance.

## Introduction

Perennial ryegrass (*Lolium perenne* L.) is an important forage grass species due to its productivity and high forage quality. However, with changing climates, improving its resistance to abiotic stresses is important to sustain grassland production. Frost is one of the abiotic stresses causing serious concern to the growth of perennial ryegrass (Galiba et al., 2009). Coping with abiotic stress is a multifaceted task that requires physiological adaptations at all levels of the organism (Sandve et al., 2011). Several quantitative trait loci (QTL) and candidate genes for freezing tolerance have been identified in perennial ryegrass and the closely related species meadow fescue (Yamada et al., 2004; Turner et al., 2006; Xiong et al., 2007; Rudi et al., 2011; Alm et al., 2011). Still, differential responses of cultivars to variable environmental conditions are genetically based, and other QTL/genes need to be identified in order to explore variation in freezing tolerance among cultivars and genotypes.

Classical linkage mapping using bi-parental mapping populations have been successful in detecting QTL and candidate genes for freezing tolerance, especially in inbreeding species like barley (Reinheimer et al., 2004) and triticale (Liu et al., 2014). Such mapping populations suffer from low resolution in detecting QTL (small population size), and the fact that only small proportions of the genetic diversity, i.e., only two alleles at a given locus in bi-parental crosses with inbred parents and up to four alleles with crosses of completely heterozygous outbreeding parents, are captured. In addition, self-incompatibility and severe inbreeding depression is common in forage grass species, thus recombinant inbred lines, which would be advantageous for QTL mapping, cannot be developed and utilized. Populations for QTL mapping in perennial ryegrass have mainly been pseudo-F2 populations from crosses between heterozygous parents (Xing et al., 2007). Association mapping, also known as linkage disequilibrium (LD) mapping, has improved mapping resolution by taking advantage of historical LDs and large population sizes. However, association mapping in plants is complicated by population structure, which is common in plant populations (Flint-Garcia et al., 2003).

Linkage disequilibrium is a non-random association of alleles between two or more linked loci. The degree of LD in any given population is dependent on (i) the reproductive biology of the organism (i.e., outbreeding vs. inbreeding) and (ii) population history. Inbreeding plant species have high LD-levels due to high levels of homozygosity, with non-random associations of alleles spanning large distances. In worldwide accessions of the inbreeding model plant, *Arabidopsis thaliana*, LD was estimated to span between 250 and 10 kb (Nordborg and Tavare, 2002; Nordborg et al., 2002, 2005; Kim et al., 2007) and in rice between 100 kb to 200 kb (Huang et al., 2010). Studies in perennial ryegrass have found various rates of LD decay. LD decayed within less than 0.5 kb in 11 disease resistance genes among 20 diverse genotypes (Xing et al., 2007), and between 0.5 and 3 kb in herbage nutritive quality genes among diverse germplasm (Ponting et al., 2007). However, significant LD extended as long as 1.6 Mb in a perennial ryegrass cultivar originating from six related parents but less than 174 kb in a cultivar originating from 336 parents (Auzanneau et al., 2007).

Knowledge of the LD structure of populations can be exploited for different purposes. If the aim is to identify causative polymorphisms underlying phenotypic variation, low LD ensures that the resolution of a marker-trait association is limited to a small section of the chromosome (e.g., within the length of a gene). This type of study must utilize either extremely high marker density (if the markers are randomly chosen) or a set of carefully selected markers which have high likelihood of being close to causative polymorphisms (i.e., markers in candidate genes) (Andersen and Lubberstedt, 2003). Conversely, if we are only interested in finding a genetic marker and use it for breeding purposes (i.e., marker assisted selection), moderate to high LD-levels plays to our advantage, as relatively high LD-levels identify marker-trait association, even if the marker is not situated in physical close proximity to the causative polymorphism. This renders it possible to identify population specific genetic markers with relatively little effort.

Understanding the genetic control of freezing tolerance would aid in the development of cultivars of perennial ryegrass with improved adaptation to frost. As part of the EU project ‘GRASP’, 20 heterozygous genotypes (Lolium Test Set, LTS) of diverse origin were assembled for identification of single nucleotide polymorphisms (SNPs) in candidate genes for specific traits and development of SNP assays for candidate gene allele tracing and validation in selection experiments (Lübberstedt et al., 2003; Posselt et al., 2006; Xing et al., 2007). Nucleotide diversity and LD in candidate genes for disease resistance and shoot morphology among the 20 LTS genotypes were investigated by Xing et al. (2007) and Brazauskas et al. (2010), respectively. Genetic differentiation in response to selection for water-soluble carbohydrate (WSC) content was reported based on divergent selections for two generations from a synthetic population established by pair-crosses among four of the LTS genotypes with contrasting WSC content (Farrar et al., 2012; Gallagher et al., 2015). A second synthetic population was established by pair-crosses among five LTS genotypes with contrasting vernalization requirements, and this population was used in the present investigation to study genetic differentiation in response to selection for freezing tolerance.

Our objectives were to: (1) test whether there are genetic variation for freezing tolerance in the synthetic population by studying responses to phenotypic selection; (2) study how divergent selection for freezing tolerance affects levels of allelic diversity, genetic structure and LD; and (3) identify candidate genes responding to selection for freezing tolerance that can be useful in breeding for freezing tolerance.

## Materials and Methods

### Plant Material and Selection for Freezing Tolerance

**FIGURE 1:**
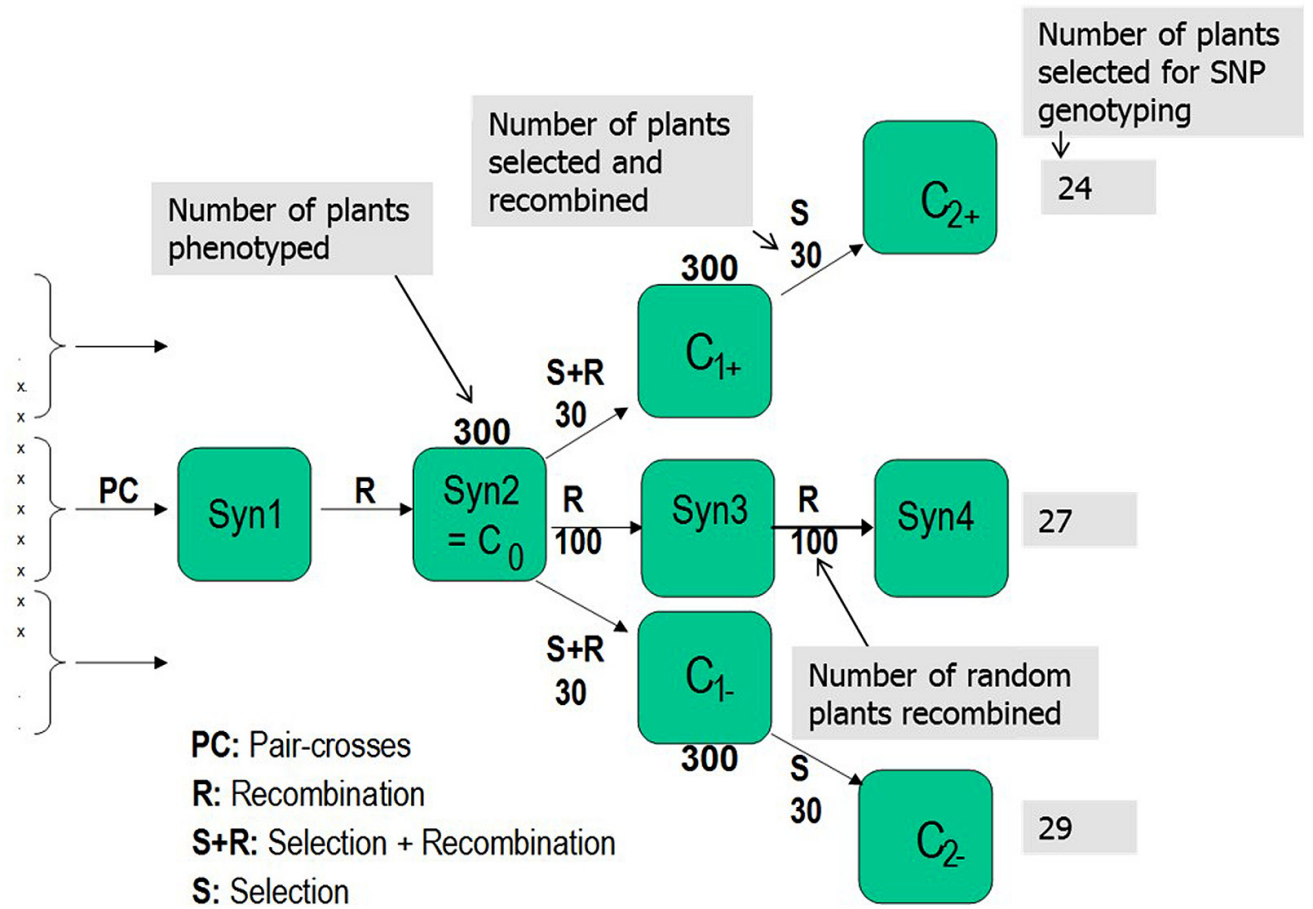
**Selection scheme employed to conduct the divergent selections for freezing tolerance in *Lolium perenne*. L.** Pair-crosses was performed among the five European *L. perenne* genotypes (LTS3, LTS4, LTS11, LTS15, and LTS16). Numbers associated with each box represent number of genotypes phenotyped in each generation and selected/recombined in each selection.

A synthetic population was made by pair-crossing five LTS genotypes (LTS 3, LTS 4, LTS 11, LTS 15, and LTS 16) in all pairwise combinations, and mixing equal amounts of seeds from each cross to establish the Syn1 population (**Figure 1**). LTS 3 and LTS 4 are the Falster (vernalization sensitive) and Veyo (vernalization insensitive) genotypes, respectively, grandparents of the Danish VrnA mapping population (Jensen et al., 2005). LTS 11 is a colchine induced rhizomatous mutant genotype from Lithuania, while genotypes LTS 15 and LTS 16 are from ecotypes from Greece and Sweden, respectively. Details about the five parental LTS genotypes can be found in Posselt et al. (2006). Due to time constraints, it was not possible to test the level of freezing tolerance among the five LTS genotypes before the experiment started. However, as the genotypes represent adaptations to a range of climatic conditions across Europe we expected that the synthetic population would contain sufficient genetic variation for freezing tolerance.

The Syn2 generation was produced from Syn1 by open pollination in isolation. Three hundred randomly selected individual plants from Syn2, hereafter termed C_0_, comprised the initial experimental population (**Figure 1**). The 300 genotypes were cloned in several ramets; some ramets were used for freezing tests and some were vernalized during autumn/winter and used to establish the divergent selections and the random mating, non-selected Syn3 population by intercrossing the following summer in pollen-proof isolation greenhouse chambers. Freezing tests of Syn2, C_1+_ and C_1-_ were conducted as described by Larsen (1978) and Alm et al. (2011) with subsequent divergent phenotypic selection for freezing tolerance. In the freezing test of the Syn2 population, replication was obtained by using six ramets of each genotype, while the C_1+_ and C_1-_ populations were tested using four ramets and the LTS genotypes by testing 12 ramets of each genotype. A selection intensity of 10% was used with 30 genotypes selected out of 300 for each round of recombination in both directions, creating the first generation high (C_1+_) and low (C_1-_), and the second generation high (C_2+_) and low (C_2-_) freezing tolerance populations. In order to quantify the effect of genetic drift, 100 randomly selected genotypes among the 300 C_0_ genotypes were intercrossed to make Syn3 seeds, from which 100 randomly selected individuals was selected among 300 individuals and recombined to make Syn4 (**Figure 1**). Twenty-four, 29 and 27 genotypes were randomly selected from the second generation high (C_2+_), low (C_2-_), and US Syn4 population, respectively, and used for SNP genotyping (**Figure 1**). In the following presentation, the C_2+_ selection is termed high-frost (HF) tolerance, the C_2-_ is termed low-frost (LF) tolerance and Syn4 is termed US (**Figure 1**).

### DNA Isolation and SNP Genotyping

For SNP genotyping, genomic DNA was extracted from the leaves of a total number of 80 genotypes representing the three populations. About 100 mg of leaf tissues were crushed with a pestle and mortar in liquid nitrogen and used for extraction of genomic DNA using a Qiagen DNeasy Plant Mini Kit (Qiagen Cat. no. 69106) following the manufacturer’s instructions (QIAGEN, Hilden, Germany). The DNA quality was assessed and normalized using the Nanodrop ND-1000 Spectrophotometer (Thermo Scientific, USA).

Two hundred and seventy-eight *L. perenne* genic SNP markers distributed across the seven linkage groups [LG1 (37); LG2 (57); LG3 (32); LG4 (40); LG5 (39); LG6 (37); and LG7 (36)] were selected from the 768 Illumina GoldenGate assay based SNP markers (Studer et al., 2012). The distribution of the selected SNPs and their inter-marker distances were mapped across the linkage groups using the MapDraw software (Liu and Meng, 2003) (**Supplementary Figure S1**). These Illumina GoldenGate assay based SNPs were converted to Sequenom MassARRAY iPLEX platform (Gabriel et al., 2001) SNPs that fall within a single exon and have sufficient flanking exonic sequence (200–250 bp) for primer design. The MassARRAY iPLEX system is based on a region-specific PCR followed by an allele specific single base extension, where products are analyzed in terms of their masses by matrix-assisted laser desorption/ionization time-of-flight mass spectrometry (MALDI-TOF MS) (Jurinke et al., 2004; Mollinari and Serang, 2015). The differences in the mass are automatically translated by the software into specific genotype calls. In addition, the Sequenom MassARRAY iPLEX platform is feasible for targeted SNP genotyping with small sample numbers rather than performing random genotyping by sequencing, which would be expensive and might not give the 278 specific SNPs used in these studies.

High-throughput genotyping was performed using the Sequenom MassARRAY iPLEX platform (Sequenom, San Diego, CA, USA) at the Centre for Integrative Genetics (CIGENE), Norwegian University of Life Sciences, Norway. Briefly, multiplex assays were designed using the MASSARRAY^®^ Assay Design software for the 278 SNPs across 12 multiplex panels set with the following parameters: amplicon length (bp): min:80, optimum:100, max:120; PCR primer length (bp): min:16, optimum:20, max:25; extension primer length (bp): min:16, max:28; hybridization Tm (°C): min:45, max:65. PCR reactions were performed using Sequenom iPLEX gold reagent kits following standard procedures (**Supplementary Tabel S1**). Approximately 20 ng of genomic DNA was amplified using a pool of 278 pairs of PCR primers under cycling conditions at 95°C for 15 min, 46 × (95°C for 25 s, 57°C for 30 s, 72°C for 70 s), and final extensions at 72°C for 3 min.

### Genetic Diversity and Analysis of Molecular Variance (AMOVA)

The set of SNPs was filtered in order to perform molecular diversity analyses. Markers with more than 10% missing genotypes and with minor allele frequency (MAF) <5% were removed. SNP genotyping data obtained after filtering was used for calculating expected (H_e_), and observed heterozygosity (H_o_) using the ARLEQUIN software 3.5.1.3 (Excoffier et al., 2005). Principal coordinate analysis (PCoA) based on a dissimilarity matrix was performed for population differentiation by the GenAlEx software version 6.5 (Peakall and Smouse, 2012). Analysis of Molecular Variance (AMOVA) was performed to estimate the variance between populations and among genotypes within populations using the ARLEQUIN software 3.5.1.3 (Excoffier et al., 2005).

### Population Structure Analysis

The “Linkage” model based approach in an ancestry model, together with a correlated allele frequency model implemented in STRUCTURE software version 2.3.4 (Pritchard et al., 2000) was used to infer the population structure. In the linkage model, a subset of markers, which are outliers and closely linked markers with a distance of less than 2 cm were excluded from the analysis. Initial STRUCTURE runs were carried out with a length of burn-in of 10,000 and MCMC (Markov Chain Monte Carlo) of 50,000. Ten independent simulations were conducted allowing *K* (number of subpopulations) to vary from 1 to 10. Once inferring the most likely *K*, we implemented more stringent parameters with a length of burn-in and MCMC (Markov Chain Monte Carlo) of 200,000 each with ten independent simulations of *K* varying from 1 to 5. The best *K* was determined by the log likelihood of the data (LnP(*D*)) in the STRUCTURE output and an ad hoc statistic Δ*K* based on the second-order rate of change in LnP(*D*) between successive *K* values (Evanno et al., 2005) using Structure Harvester (Earl and Vonholdt, 2012) and CLUMPAK (Kopelman et al., 2015).

### Linkage Disequilibrium Analysis

Genome-wide LD analysis was performed in each of the three population groups (HF, LF, and US) by pairwise comparisons among the SNP markers distributed across seven linkage groups using the HAPLOVIEW software version 4.2 (Barrett et al., 2005) with the following parameters: MAF > 0.05; Hardy–Weinberg *P*-value cut-off, 0; and percentage of genotyped lines >0.50. LD was estimated using squared allele frequency correlations (*r*^2^) between the pairs of loci. The loci were considered to be in significant LD when *P* < 0.001, the rest of *r*^2^ values was not considered as informative. The pattern and distribution of intra-chromosomal LD was visualized and studied from LD plots generated for each chromosome by HAPLOVIEW software version 4.2. To investigate the average LD decay in the whole genome among the panel, significant intra-chromosomal *r*^2^ values were plotted against the genetic distance between markers.

### *F*_*st*_ Outlier Tests for Detecting SNP Loci under Selection

To detect loci under directional selection, we used the two coalescent-based simulation methods of Beaumont and Nichols (1996) and Excoffier et al. (2009). For the method of Beaumont and Nichols (1996), we used the program LOSITAN (Antao et al., 2008) to generate 100,000 simulated loci, providing an expected neutral distribution of *F_st_* values and an estimate of *P*-value for each locus. This method detects genes under selection based on the distributions of heterozygosity and *F_st_* (Beaumont and Nichols, 1996) by the two options “neutral mean *F_st_*” and “force mean *F_st_*” as recommended by Antao et al. (2008). Markers with *F_st_* values higher than 95% of the neutral distribution were considered to be under divergent selection, and markers with *F_st_* lower than 95% of neutral distribution were inferred to be subject to balancing selection.

The hierarchical method of Excoffier et al. (2009), which is a modification of the approach of Beaumont and Nichols (1996), was performed as implemented in the ARLEQUIN software package version 3.5.1.3 (Excoffier and Lischer, 2010). We simulated a hierarchical island model based on three groups of three demes with 50,000 simulations to generate the joint distribution of *F_st_* versus heterozygosity. Loci that fall out of the 99% confidence intervals of the distribution are identified as outliers, being putatively under selection. The putative function of genes with outlier SNPs detected by these two methods was identified using the Gene Ontology (GO) annotation using the Blast2GO software tool version 3.0 (Conesa et al., 2005).

## Results

### Phenotypic Variation for Freezing Tolerance and Response to Phenotypic Selection

**FIGURE 2:**
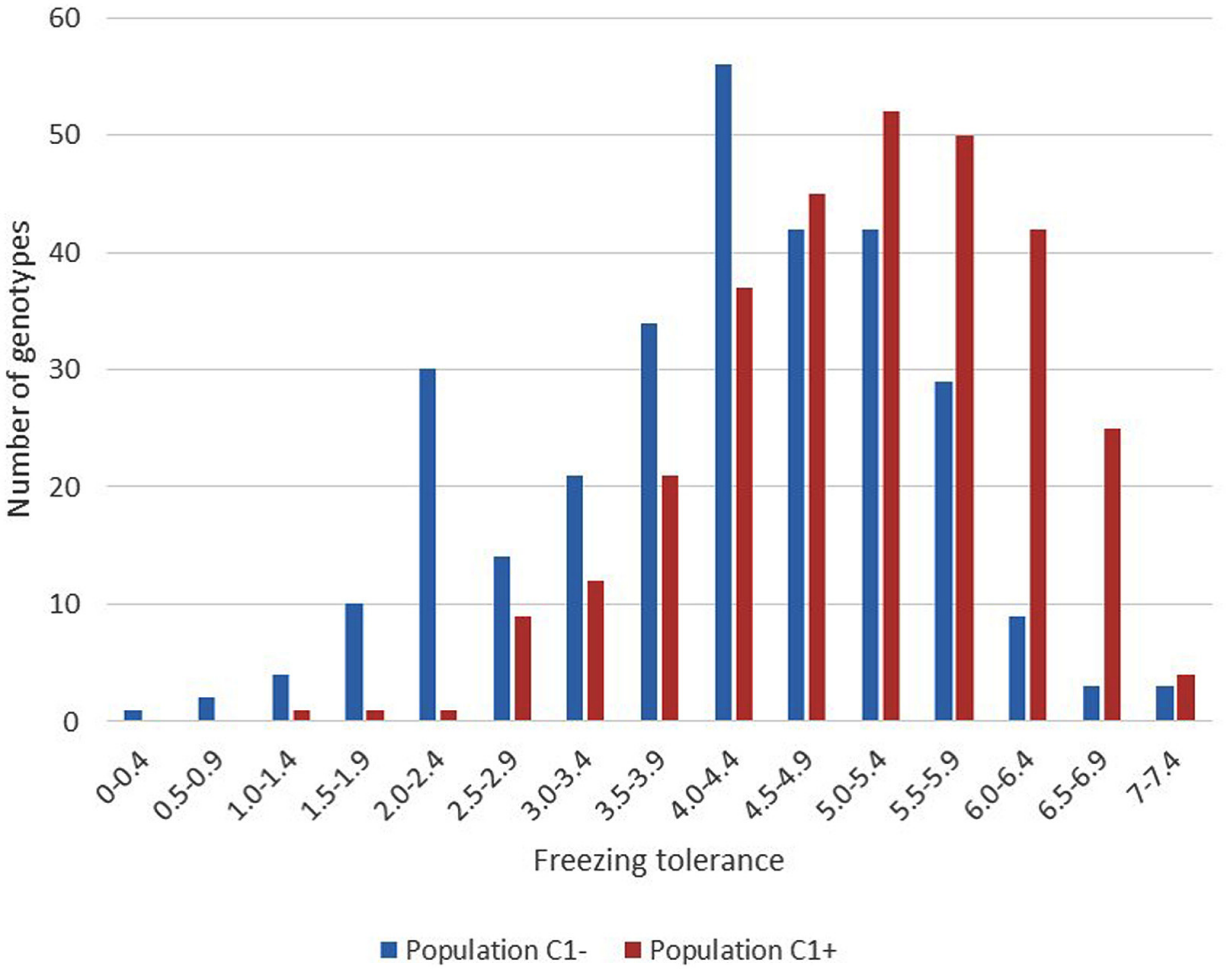
**Variation in freezing tolerance among the 300 plants within each of the divergent selections C_**1-**_ and C_**1+**_.** (Scale regrowth test after freezing 0 = completely dead, 9 = no visible injury).

The results of phenotyping the LTS genotypes, the synthetic base population (Syn2) and the divergently selected populations for low (C_1-_) and high (C_1+_) freezing tolerance are presented in **Table 1**. Visual scoring of regrowth is not comparable across experiments since it is dependent on experimental conditions, seasons, and conditions during cold acclimation of the plant material. The LTS genotypes were all tested in the same freezing test but independent of the other populations. The freezing scores for the C_1-_ and C_1+_ populations can be compared since they were obtained in the same freezing test, while the freezing scores of the Syn2 population was obtained in an earlier, independent test and is not comparable with the others. Therefore, it is not possible to use the selection differentials and the selection responses to calculate realized heritability. The broad sense heritabilities (repeatabilities) presented were calculated using the between ramet variation as estimate of the environmental variation, and the variation of genotype means as the genotypic variation (**Table 1**). The results show that there is a large variation for freezing tolerance in the base population, ranging from 0.33 to 6.47 (scale 0–9), actually very similar to the range among all LTS genotypes (0–6.38). However, the mean value in the Syn2 population was low (3.23). This is expected in view of the rather low freezing tolerance among the five LTS genotypes used to establish the Syn2 population; the four genotypes that was successfully phenotyped had an average freezing score of 4.21. Freezing tolerance is a complex trait with low heritability, and proper replication is required in freezing tests. This effect is evident in the present study. Broad sense heritabilities were 0.18 and 0.25 in the C_1+_ and C_1-_ populations, respectively, with four ramets of each genotype tested, while it increase to 0.45 in the test of the LTS genotypes which were replicated 12 times each (**Table 1**). Despite relatively low heritability of the freezing tolerance, the responses to one generation of selection were clear as evident from the different mean freezing scores of the C_1-_ and C_1+_ populations (**Table 1**). However, the distributions of the freezing scores in the two populations was nearly completely overlapping (**Figure 2**).

**Table 1:** Mean values, range, and broad sense heritability (H^2^_B_) of freezing tolerance of the synthetic base population, the divergent selections, and the LTS genotypes.

Population	*N*	Mean	LSD_0.5_	Range	Heritability	Selected high frost (HF)		Selected low frost (LF)	
						Mean	Range	Mean	Range
Syn2 (C_0_)	300	3.23	1.20	0.33–6.47		4.94	4.35–6.47	1.51	0.80–2.07
C_1+_	300	5.12	1.85	1.49–7.39	0.18	6.75	6.49–7.39		
C_1-_	300	4.17	1.99	0.48–7.33	0.25			2.61	1.67–3.47
LTS genotypes	19^1^	4.13	1.58	0.00–6.38	0.45				
LTS 03		4.68							
LTS 04		-							
LTS 11		4.79							
LTS 15		4.43							
LTS 16		2.95							

*Freezing tolerance was scored as regrowth on a scale 0 = no regrowth (dead, susceptible to freezing) to 9 = maximum regrowth (no visible injury, freezing tolerant).*

*^1^No freezing test data obtained for LTS 04 due lack of vernalization requirement, it flowered during multiplication and died during freeze testing.*

### Genotyping, Genetic Variability, and AMOVA

High-throughput genotyping was performed by Sequenom MassARRAY iPLEX using 278 *L. perenne* genic SNP markers distributed across the seven linkage groups [LG1 (37); LG2 (57); LG3 (32); LG4 (40); LG5 (39); LG6 (37); and LG7 (36)] in 80 genotypes of HF (24), LF (29), and US (27) (**Supplementary Figure S1**). Eleven SNPs failed in genotyping and 27 SNPs with missing data >5% were removed from the dataset. Of the remaining 231 SNPs, 181 SNPs (78.3%), 162 SNPs (70.1%), and 180 SNPs (77.9 %) were polymorphic across the HF, LF and US populations, respectively. Observed heterozygosity was slightly lower than expected heterozygosity in all the populations, indicating either the occurrence of null alleles or a slight level of inbreeding (**Table 2**). The F_IS_ (inbreeding coefficient) is negative in the high frost population, implying a considerable degree of outbreeding, whereas positive *F*_IS_ in the low and US populations showed considerable degree of inbreeding. The AMOVA for the three populations revealed that 91.6% (*P* < 0.014) of the genetic variation is found within individuals, whereas 8.67% (*P* < 0.0001) of the genetic variation is found among populations (**Table 3**, **Supplementary Figure S2**). In addition, AMOVA was separately conducted between HF and LF, HF and US, and LF and US populations in order to study genetic variation between populations. 13.14% (*P* < 0.0001) of the genetic variation was found between HF and LF, only 3.08 % (*P* < 0.0048) between HF and US, and 8.67% (*P* < 0.0001) between LF and US (**Table 3**).

**Table 2:** Genetic diversity of three experimental populations.

Population	*n*	*H*_0_	*H*_e_	*F_IS_*
High Frost (C_2+_)	24	0.33783	0.34032	-0.03929
Low Frost (C_2-_)	29	0.35058	0.37852	0.01731
Unselected (Syn4)	27	0.3182	0.34493	0.00471

*n = total number of genotypes; *H*_0_ = observed heterozygosity; *H*_e_ = expected heterozygosity; *F_IS_* = the inbreeding coefficient.*

**Table 3:** Results of the Analysis of Molecular Variance (AMOVA) for three experimental populations.

Group	Partitioning	df	Sum of squares	Variance components	Percentage of variation	*P*-value
Three populations (HF, LF, and US)	Among populations	2	368.174	2.8907	8.67	0.00001^∗∗∗^
	Among individuals within populations	77	2338.807	-0.09106	-0.27	0.55425
	Within individuals	80	2444.5	30.55625	91.61	0.01466^∗∗^
	Total	159	5151.481	33.35588		
HF and LF	Among populations	1	265.151	4.48947	13.14	0.00001^∗∗∗^
	Among individuals within populations	51	1495.698	-0.34573	-1.01	0.652
	Within individuals	53	1591	30.01887	87.87	0.03715^∗^
	Total	105	3351.849	34.1626		
HF and US	Among populations	1	77.831	0.95099	3.08	0.00489^∗∗^
	Among individuals within populations	49	1445.424	-0.39784	-1.29	0.61584
	Within individuals	51	1545	30.29412	98.21	0.43891
	Total	101	3068.255	30.84727		
LF and US	Among populations	1	182.732	2.74835	8.67	0.00001^∗∗∗^
	Among individuals within populations	54	1567.116	0.05944	0.19	0.48583
	Within individuals	56	1618.5	28.90179	91.15	0.07429
	Total	111	3368.348	31.70958		

*HF, high frost (C_2+_), LF, low frost (C_2-_), and US, unselected (Syn4). ^∗^*P* < 0.050; ^∗∗^*P* < 0.010; ^∗∗∗^*P* < 0.001.*

### Population Structure

**FIGURE 3:**
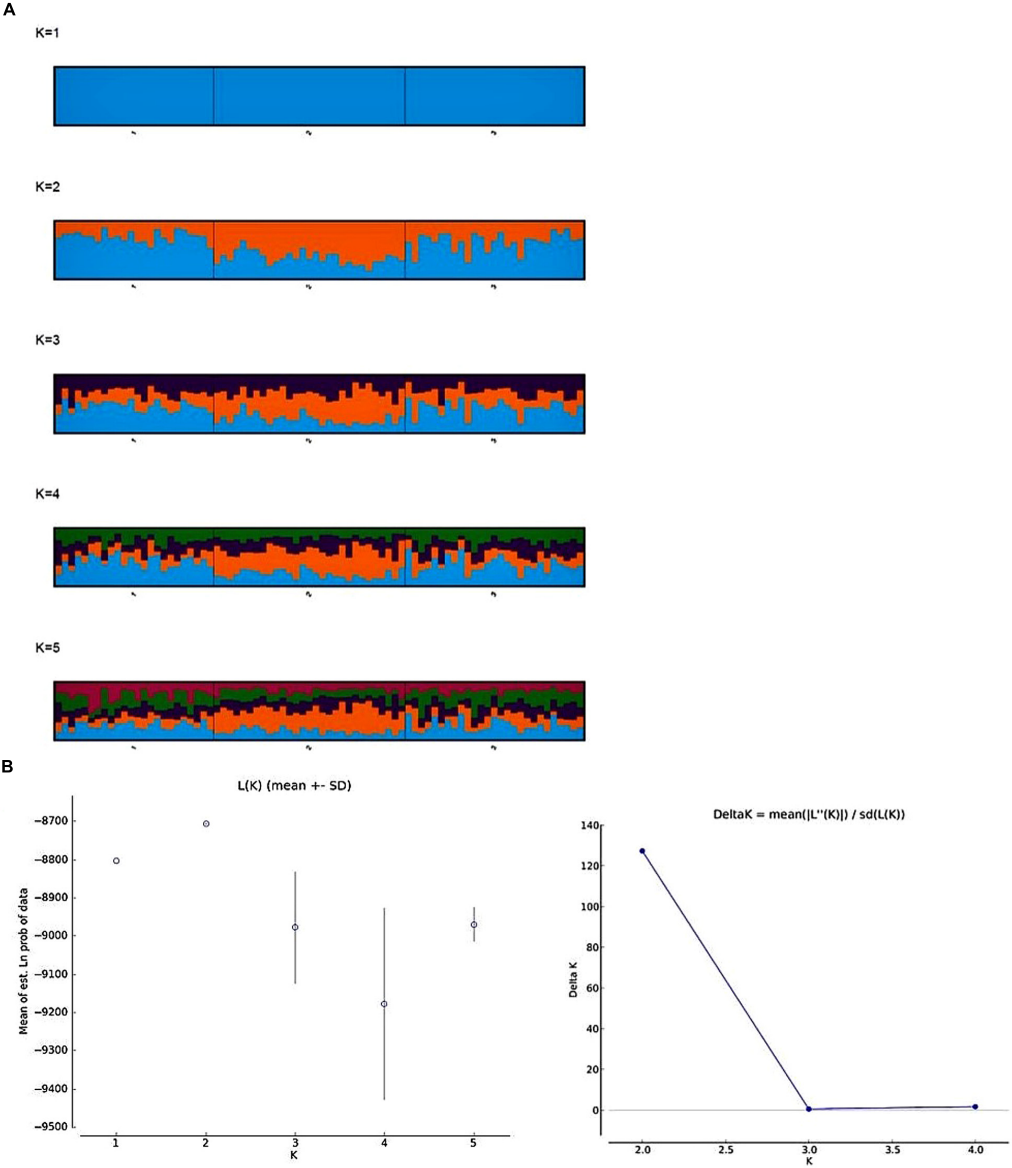
**Genetic subdivision among the three frost populations; (1) high, (2) low, and (3) unselected. (A)** Bar plots from CLUMPP results aligning 10 structure runs for *K* = 1 to 5 with an iteration of 10 for each run. Each bar plot accompanied with its statistical *K* by Pritchard’s (Pritchard et al., 2000) and Evanno’s method (Evanno et al., 2005). The plots are read from left to right, with bars representing individuals and the color of the bar representing the proportion of individual markers that originated from certain population. **(B)** Estimated log likelihood of the data (LnP(D)) and ad hoc Δ*K* over 10 repeats of STRUCTURE analysis for *K* = 1–5 subpopulations.

To infer the population structure from multilocus genotype data among the three populations, we implemented the linkage model in the STRUCTURE software. Overall, this model retains the main elements of the admixture model and reports the overall ancestry for each individual, taking account of the linkage. However, the model is not designed to deal with background LD between very tightly linked markers (Pritchard et al., 2000). Hence, we discarded 94 SNP markers closely linked with a minimum distance of 2 cm and 32 outlier SNP markers from the analysis. In total, 105 SNP markers were used to estimate the population structure. The LnP(*D*) value for each given *K* (number of subpopulations) increased from 1 to 2 and gradually decreased between 3 and 4 and slight increase in 5, showing evidence of maximum at *K* = 2 (**Figure 3B**). The second order likelihood, Δ*K* was calculated and observed the maximum Δ*K* value at *K* = 2. Both Pritchard’s and Evanno’s methods confirmed the *K*-value as 2 (**Figure 3B**), however, we plotted the barplots from *K* = 1 to 5, as LnP(*D*) value was slightly higher at *K* = 5. The barplots showed higher proportion of presumably alleles for freezing tolerant (blue) and lower proportion of presumably alleles for low freezing tolerant (orange) in HF and US populations, whereas, high proportion of orange and low proportion of blue was observed in LF population. This pattern was observed consistently from *K* = 2 to 5 (**Figure 3A**).

**FIGURE 4:**
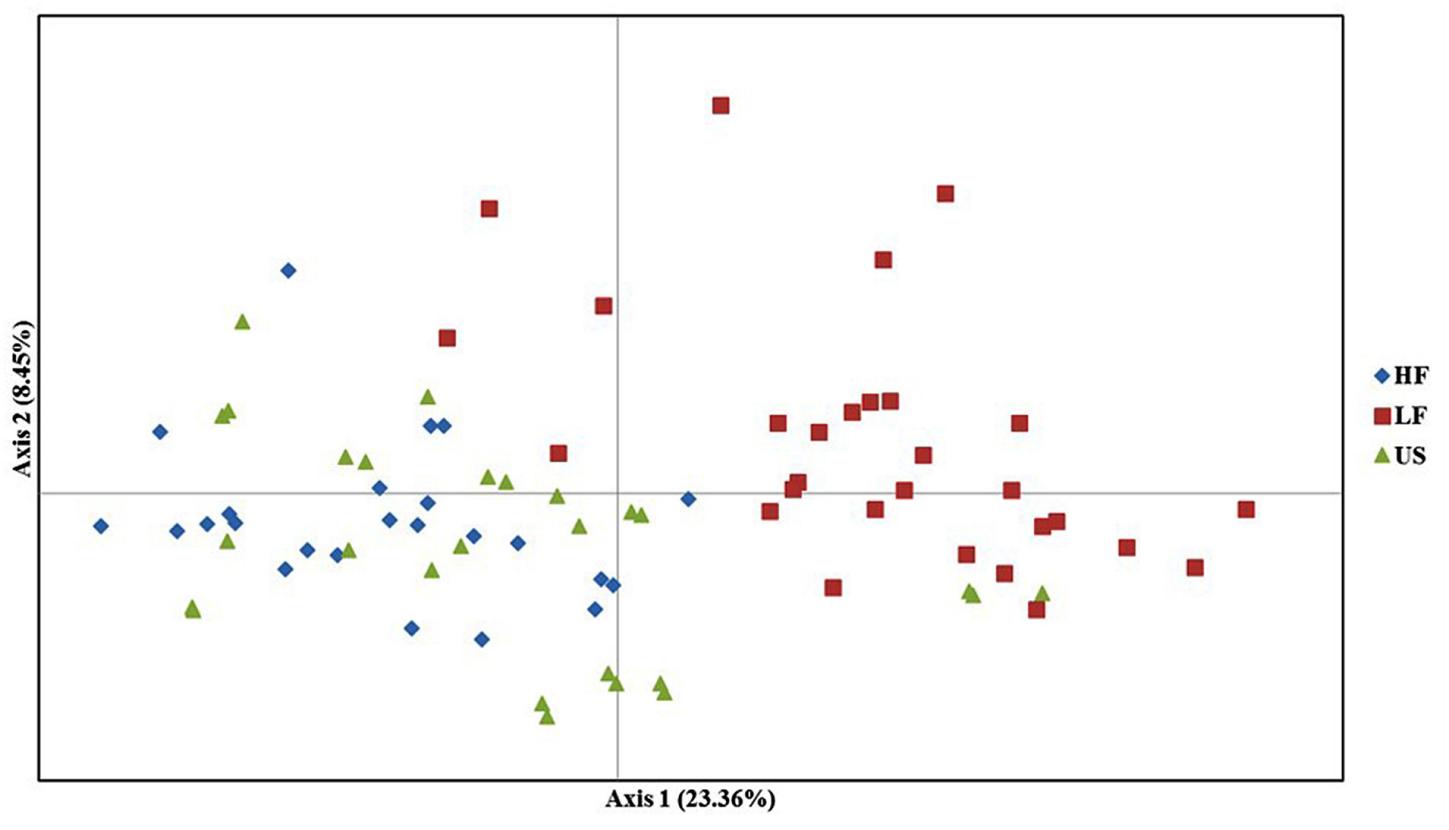
**Principal coordinate analysis (PCoA) of high frost (HF), low frost (LF), and US populations.** The different colors represent the three populations. The first and second principal coordinates account for 23.6 and 8.45% of total variation, respectively.

The PCoA based on the dissimilarity matrix clearly separates the HF and LF populations, whereas the US population overlaps nearly completely with the HF population. The first and second principal coordinates explained 23.36 and 8.45% of the molecular variance (**Figure 4**).

### Linkage Disequilibrium

**FIGURE 5:**
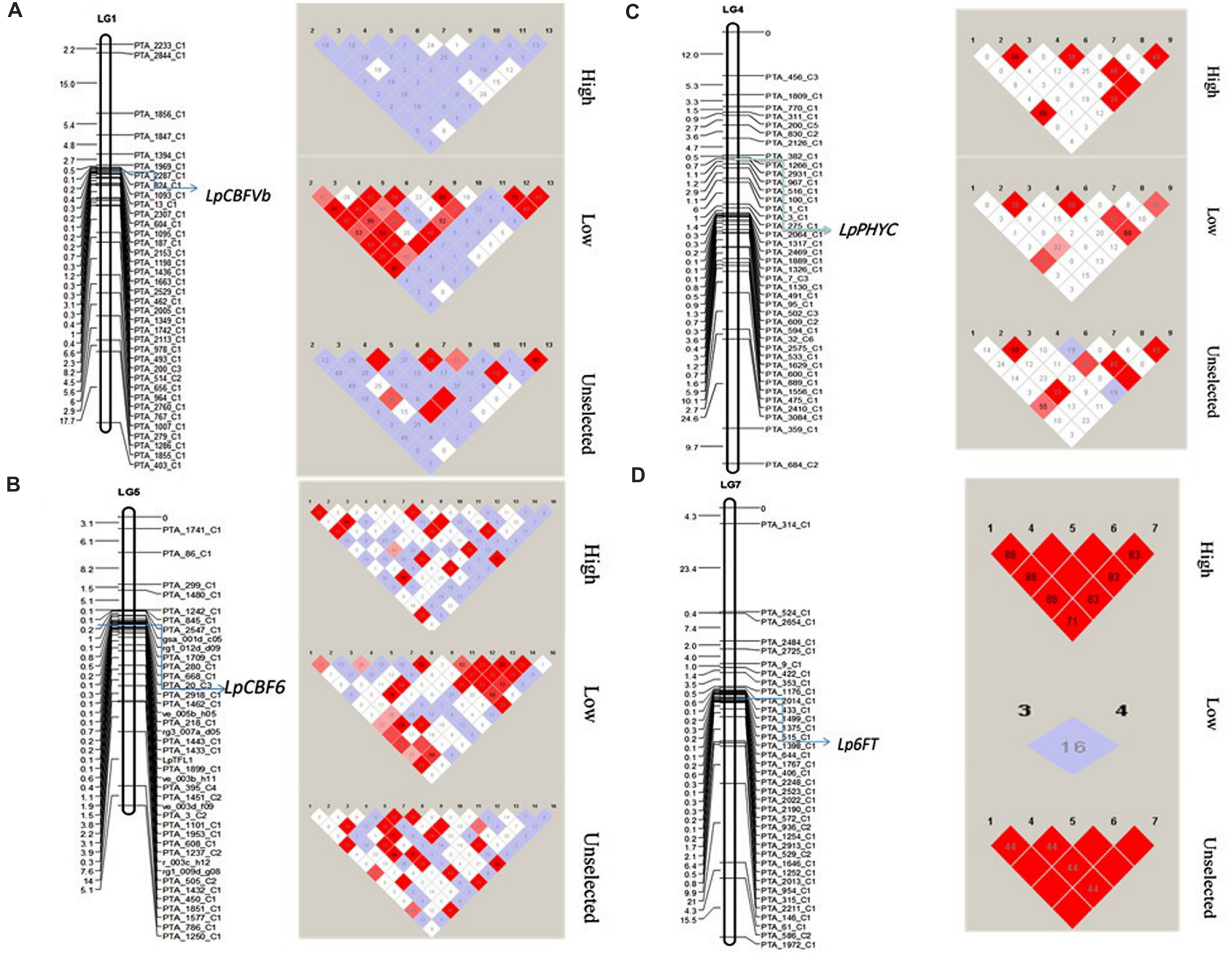
**Linkage disequilibrium among the three experimental populations (HF, LF, and US) across four important frost related genes **(A)***LpCBFVb*, **(B)***LpPHYC*, **(C)***LpCBF6*, and **(D)***Lp6FT*.** Left side maps shows the location of the gene and the markers on linkage groups. Right side shows the LD distribution. Red blocks denote high LD.

A random subset of markers spanning across all seven chromosomes (see Materials and Methods) of *L. perenne* was used to calculate LD decay. Interalleleic *r*^2^ values (association between any pairs of alleles from different loci) were calculated after removing the low frequency alleles (<0.05). The *r*^2^ values for the linked loci were plotted against the genetic distance to observe the LD decay (**Supplementary Figure S3**). Slow LD decay was observed on chromosomes 4 and 7, exceeding 90 cM, whereas rapid LD decay was observed in chromosome 5, below the significant threshold (*r*^2^ ≥ 0.03) within 0.5 cM. However, the pattern of LD decay varied between HF, LF, and US across the chromosomes. LF has low decay in chromosomes 2, 4, and 6 with significant threshold (*r*^2^ ≥ 0.10), compared to HF and US. In addition, we also observed the LD around the important frost candidate genes *LpCBFVb* (Chr.1), *LpPHYC* (Chr. 4), *LpCBF6* (Chr.5), and *Lp6FT* (Chr.7) between HF, LF, and US populations (**Figure 5**). No LD was observed at *LpCBFVb* for HF, whereas very high LD was observed in LF with several tightly linked markers, and moderate LD was observed in US with few tightly linked markers. LD at *LpPHYC* was almost similar in the three populations. LD at *LpCBF6* was high in US and LF, but low in HF. At *Lp6FT*, very high LD was observed in HF and US, where all five SNP markers are in high LD, but no LD was observed in LF (**Figure 5**).

### Loci under Selection

**FIGURE 6:**
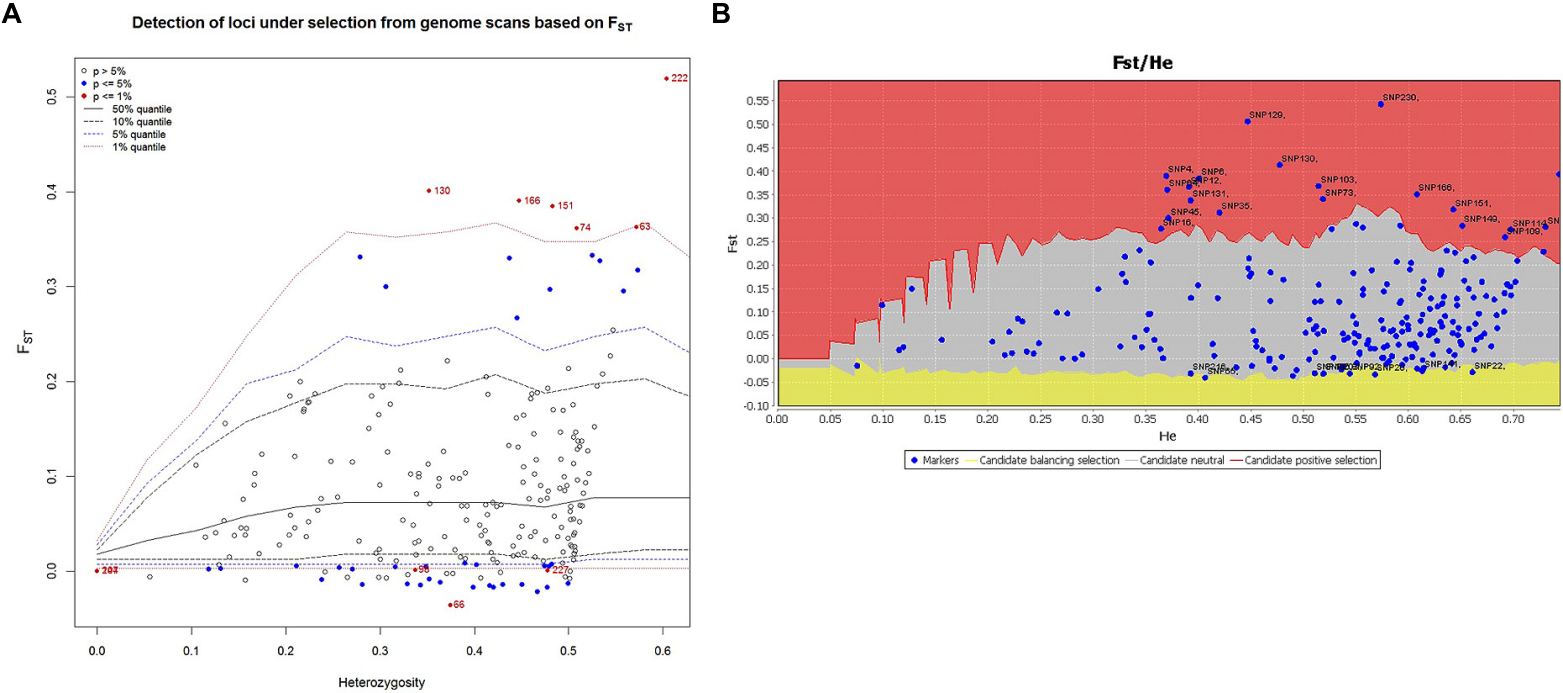
**Candidate loci under selection were identified using two *F_st_* based outlier approaches. (A)** Hierarchical structure model using Arlequin 3.5. *F_st_*: locus –specific genetic divergence among the populations; Heterozygosity: measure of heterozygosity per locus. Loci significant at the 1% level are indicted by red dots. **(B)** Finite island model (fdist) by LOSITAN. Loci under positive selection above 95% percentile (red area), neutral loci (gray area) and loci under balancing selection (yellow area).

Signatures of directional and balancing selection were identified at 41 loci among the three populations using the programs LOSITAN and ARLEQUIN (**Figure 6**). Putative balancing selection was detected at 15 loci (**Figure 6**). Directional selection was detected for 20 loci by LOSITAN and 6 loci by ARLEQUIN (**Figure 6**; **Table 4**). Although footprints of directional selection were differently identified for some loci with these two outlier methods, six loci were indicated to be under directional selection by both hierarchical methods employed in ARLEQUIN and LOSITAN. Putative functions of these loci are described in **Table 4**.

**Table 4:** Candidate single nucleotide polymorphisms (SNPs) under selection for freezing tolerance.

SNP ID	Chromosome	Gene function
PTA_817_C1	2	Elongation factor G-2, chloroplastic
PTA_1219_C2	3	60S ribosomal protein L37-2-like
PTA_475_C1	4	ATP phosphoribosyltransferase
PTA_1433_C1	5	40S ribosomal protein S29
PTA_450_C1	5	Beta 1,6-glucanase
PTA_1254_C1	7	Vesicle-associated protein 4-1-like

## Discussion

### Genetic Structure and Variation in Three Populations

In our studies, observed heterozygosity was slightly lower than expected heterozygosity in all the populations indicating a minor level of inbreeding (**Table 2**). These results are consistent with the genetic diversity studies of the same LTS genotypes for WSC content (Gallagher et al., 2015) and for disease resistance (Xing et al., 2007). In theory, open pollination should ensure random mating (panmixis) between all individuals in the parent population. However, some level of assortative mating, especially during crossing to produce the Syn2 and the divergently selected populations, is likely due to differences in pollen production, variation in flowering time and self-incompatibility alleles. Contrary to Gallagher et al. (2015), we did not observe any reduction in heterozygosity levels, which could have been expected after strong selection (**Table 2**; Remington et al., 2001; Beissinger et al., 2014).

Although we did not test the HF (C_2+_), LF (C_2-_), and US (Syn4) populations for freezing tolerance, we see effect of selection already in the first generation of high (C_1+_) and low (C_1-_) populations (**Figure 2**) and we expect that there has been further differentiation in the following generation. The effect on genetic differentiation was largest in the LF population (negative selection). The LF population is almost entirely separated from HF and US populations in the PCO (**Figure 4**), and in the Structure analysis with *K* = 5 we see that the population is dominated by one of the five parental genotypes (orange in **Figure 3**). One of the parental genotypes, LTS16, has markedly lower freezing tolerance than the others (**Table 1**) and this genotype most likely contributes more to the LF population than the other parental genotypes do. Individuals with higher freezing tolerance in C_0_ and C_1-_ probably have more of the genomes from the other, more freezing tolerant parents, and they will be selected against. Moreover, contributions from LTS16 is probably selected against in the HF population, making the HF and LF populations highly differentiated (**Table 3**). Remarkably, in spite of phenotypic divergence there is little genotypic divergence between the HF and US populations. Only 3.08% of the variation is found between the groups (in contrast to the 8.675% found between LF and US) and there is nearly complete overlap between the HF and US in the PCO plot (**Figure 4**). The parental genotypes LTS03, LTS11 and LTS15 all have medium levels of freezing tolerance, however, they vary in their genetic background and probably harbors variation for freezing tolerance resulting in approximately equal contribution (probably also from LTS04 and to some extent LTS16, see **Figure 3**) to the HF population. Consequently, divergence from US will be minimal. This is opposite to what was found in Gallagher et al. (2015) who identified larger differentiation between the population selected for high WSC content (positive selection) and the US population compared to the population selected for low WSC content (negative selection) and the US population.

### Linkage Disequilibrium

In synthetic populations/cultivars based on few founder genotypes, we expect very high LD compared to natural populations. Auzanneau et al. (2007) showed that after three generations of random mating, LD in synthetic populations based on contrasting numbers of founding parental genotypes varied from 174 Kb in a synthetic cultivar based on 336 founding individuals to 1.4 Mb in a synthetic based on six related individuals. These LD estimates were established using a few SSR markers (6) and a sequence variation in a candidate gene (GAI). In the present study, we used five founder genotypes and employed relatively strong selection pressures for two generations. The selection regime reduces effective population size, which leads to reduction in genetic variation and thus an increase in LD in the selected populations. This should create extensive LD, which is also evident from our results. LD observed in the selected populations in this study was substantially higher than observed by Auzanneau et al. (2007) in populations of comparable founder sizes.

We also observed high LD for the HF and US populations in *Lp6FT*, for the low frost population in *LpCBFVb* (**Figure 5**), similar to the previous studies on the gibberelic acid insensitive gene (GAI) region in a collection of 47 Eurasian ryegrass ecotypes (Auzanneau et al., 2007), and the *LpHD1* gene associated with flowering time in perennial ryegrass (Skot et al., 2007). One possible explanation for this high LD, also mentioned by Brazauskas et al. (2011), is that strong directional selection and population bottlenecks might have created islands of long-range LD in the genome of perennial ryegrass. Another explanation is that if there are segregating alleles/QTL in the populations with strong associations with freezing tolerance, it is likely that long-range haplotypes or whole chromosomes are selected for or against, and this creates strong LD. This might be the reason for the high LD found on chromosome 4 where the vernalization gene (*LpVRN1*) is located (Jensen et al., 2005), since two of the parental LTS genotypes have very different vernalization requirements. In *Festuca pratensis* it has been shown that *FpVRN1*, located in a syntenic position to *LpVRN1* on LG4 is very close to (or is itself) a QTL for freezing tolerance (Alm et al., 2011).

The LD decay varied across chromosomes in the three populations (**Supplementary Figure S3**). Similar interchromosomal heterogeneity in LD was observed in rice (Mather et al., 2007), loblolly pine (Brown et al., 2004) and in pigs (Nsengimana et al., 2004). One explanation could be the ‘Bulmer effect’ describing a higher LD between genomic regions harboring QTL undergoing selection (Bulmer, 1971). Another explanation for different rates of decay in LD between chromosomes is that the precision of linkage map distances differs between chromosomes because of different number of markers used, marker variability, and genotyping errors (Li and Merila, 2010). However the overall LD decay close to *r*^2^< 0.1 within a length of 0.5 cM is similar to previous studies using populations of diverse *L. perenne* genotypes (Ponting et al., 2007; Xing et al., 2007).

### Signatures of Selection for Freezing Tolerance

*F_st_* outlier approaches have been applied to many crops, e.g., tomato (Sim et al., 2011) and common bean (Papa et al., 2007) for identifying adaptive differentiation. Markers detected in these crops were mapped to genomic regions with known QTL/genes related to domestication. In the present study, we employed two *F_st_* outlier methods; finite island model (fdist) by LOSITAN and hierarchical structure model using Arlequin to detect true positive loci under selection (**Figure 6**). Tsumura et al. (2012) used four different *F_st_* outlier models to weed out false positive loci in conifers.

The present study identified six candidate loci (2.59 %) under positive selection based on *F_st_* values that displayed differentiation higher than the 99% limit of the confidence intervals (**Figure 6**; **Table 4**). These six loci, i.e., PTA_817_C1, PTA_1219_C2, PTA_475_C1, PTA_1433_C1, PTA_450_C1, and PTA_1254_C1 may be directly under selection. Annotation of these SNP sequences revealed the putative functions of all these six candidate loci (**Table 4**). PTA_817_C1 was identified as an elongation factor G-2, chloroplastic which might be involved in freezing tolerance. A previous transcriptome study of barley leaves revealed that the induction of several elongation factors and the chloroplast function in the leaf were the key differences between a frost tolerant and a frost-susceptible cultivar (Janska et al., 2014). Two candidate SNP loci, PTA_1219_C2, and PTA_1433_C1, a 60s ribosomal protein L37-2 like and 40S ribosomal proteins S29, respectively, might be involved in cold adaptation. The role of ribosomal proteins in *de novo* protein synthesis necessary for cold response and for the integrity of the translation machinery, which is an important factor for cold acclimation, have been demonstrated by Kim et al. (2004).

PTA_1254_C1 was identified as a vesicle-associated protein 4-1 like possibly involved in abiotic stress. Genes involved in vesicle transport have been studied extensively (Mazel et al., 2004; Ma and Bohnert, 2007). Over-expression of *AtRab7*, a gene involved in regulation of vesicle trafficking, increased endocytosis in roots, as well as salt and osmotic stress tolerance (Mazel et al., 2004). This indicates the importance of regulated vesicle trafficking for acquisition of abiotic stress tolerance.

## Conclusion

The selection scheme employed here by establishing an experimental synthetic population from five diverse *L. perenne* genotypes and selecting divergently for freezing tolerance has been successful in producing phenotypic and genotypic divergences. We found that the population selected for HF tolerance and the population selected for LF tolerance are very divergent, whereas HF and the unselected (US) control population are most similar. LD decay varied across the seven chromosomes and patterns of LD between the HF and the LF population are signs of divergence relative to freezing tolerance. Finally, six candidate loci detected independently by two *F_st_* outlier methods were candidates for loci being under directional selection for frost and might be potential marker resources for breeding perennial ryegrass cultivars with improved freezing tolerance.

## Data Accessibility

Single nucleotide polymorphisms genotyping raw data and processed data are deposited in DRYAD Digital Repository along with the input files for running Arlequin and Structure softwares (http://datadryad.org/resource/doi:10.5061/dryad.sd1dt).

## Conflict of Interest Statement

The authors declare that the research was conducted in the absence of any commercial or financial relationships that could be construed as a potential conflict of interest.

## Supplementary Material

-1.8pcThe Supplementary Material for this article can be found online at: http://journal.frontiersin.org/article/10.3389/fpls.2015.00929

TABLE S1 **List of primers used for SNP genotyping**.

Figure S1 **Selected genic SNP markers distributed across the seven linkage groups in *Lolium perenne***.

Figure S2 **Graphical representation of relationships between the high frost (HF), low frost (LF), and unselected (US) population as described by average number of pairwise differences and a matrix of pairwise *F_st_* values. (A)** Orange–red on the diagonal corresponds to pairwise differences within populations, green above the diagonal corresponds to between population (*x* and *y*-axes), blue below the diagonal corresponds to genetic distance (*d*) between populations. **(B)** Population relatedness was determined by *F_st_* values between populations.

Figure S3 **Pattern of triangle plots depicting LD values across chromosomes using *r*^2^ in three populations (high, low, unselected).** Red blocks denote high LD. Seven rows from top to down, represents chromosomes one to seven.
