# Supplementary figures and images for: Population Structure, Genetic Variation, and Linkage Disequilibrium in Perennial Ryegrass Populations Divergently Selected for Freezing Tolerance
Source: Front Plant Sci. 2015 Nov 12;6:929. doi: 10.3389/fpls.2015.00929 (PMC4641910; doi:10.3389/fpls.2015.00929)

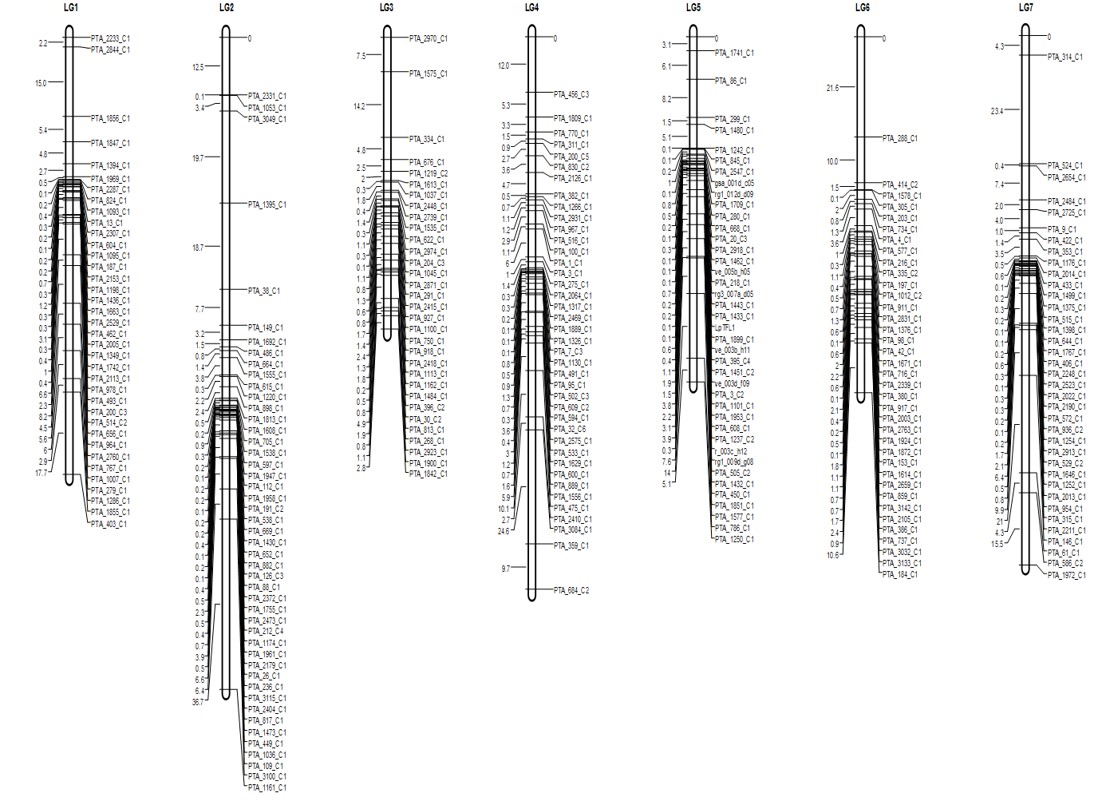

Supplement: Figure S1 — Selected genic SNP markers distributed across the seven linkage groups in Lolium perenne. [file Image_1.JPEG]

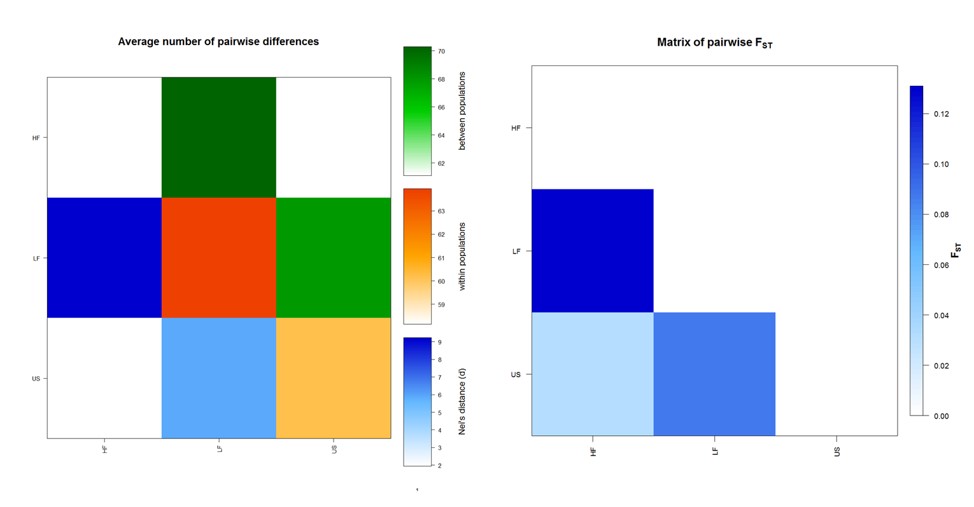

Supplement: Figure S2 — Graphical representation of relationships between the high frost (HF), low frost (LF), and unselected (US) population as described by average number of pairwise differences and a matrix of pairwise Fst values. (A) Orange–red on the diagonal corresponds to pairwise differences within populations, green above the diagonal corresponds to between population (x and y-axes), blue below the diagonal corresponds to genetic distance (d) between populations. (B) Population relatedness was determined by Fst values between populations. [file Image_2.JPEG]

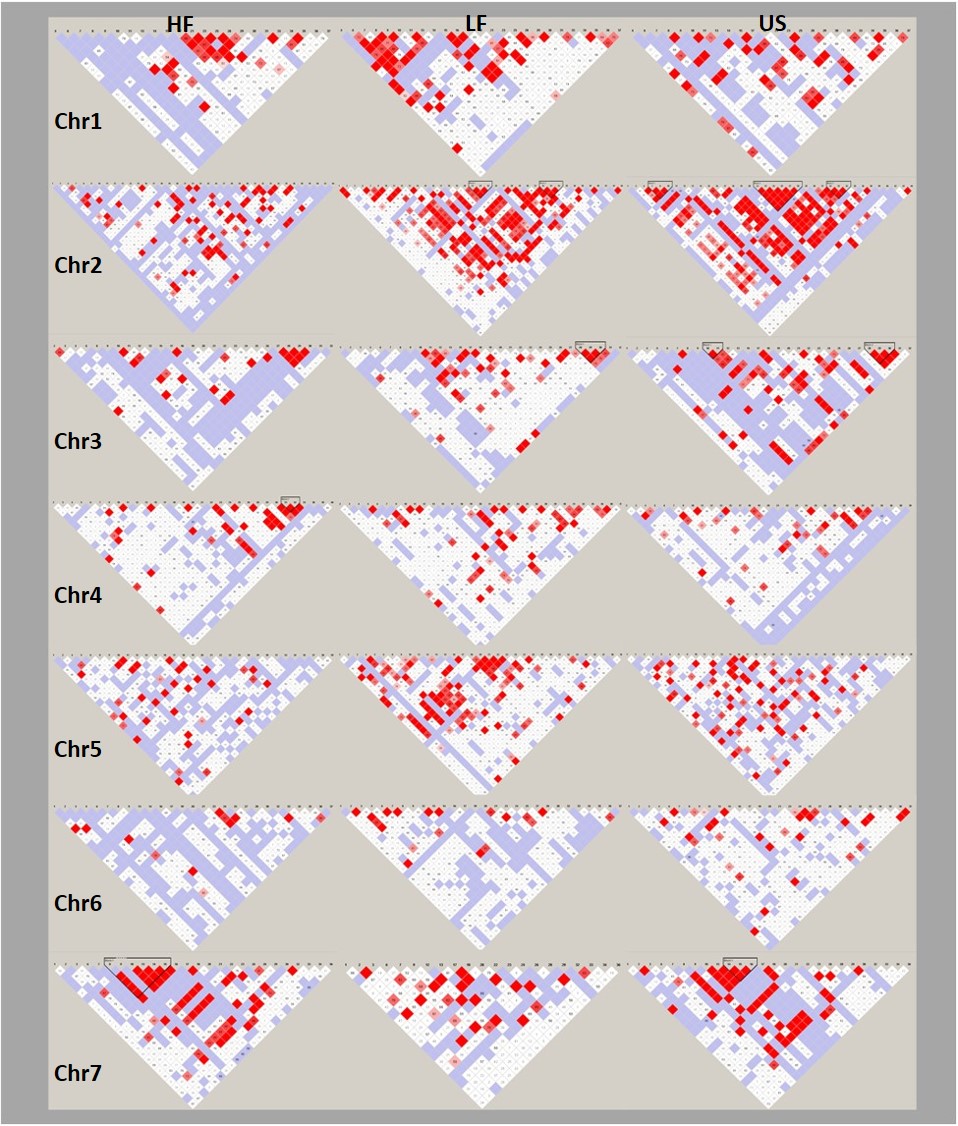

Supplement: Figure S3 — Pattern of triangle plots depicting LD values across chromosomes using r2 in three populations (high, low, unselected). Red blocks denote high LD. Seven rows from top to down, represents chromosomes one to seven. [file Image_3.JPEG]
